# And What About Exercise? Fitness and Risk of Death in “Low-Risk” Adults

**DOI:** 10.1161/JAHA.112.003228

**Published:** 2012-08-24

**Authors:** Michael S. Lauer

**Affiliations:** Division of Cardiovascular Sciences at the National Heart, Lung and Blood Institute (NHLBI) of the National Institutes of Health (NIH)

**Keywords:** editorials, exercise, risk, low, Framingham Risk Score, cardiovascular diseases

Leafing through this morning's Sunday paper, I could not help but feel a sense of unease about our current state of preventive medicine. A lead article in the opinion section argued that most of the elements of the “annual physical exam” are “in many ways pointless or (worse) dangerous.”^1^ Routine electrocardiograms are of “no use.” Another essay attacked the government's guidelines for dietary salt reduction, noting that the “eat-less-salt argument has been surprisingly controversial…Not because the food industry opposes it, but because the evidence to support it has always been so weak.”^2^ Yet another writer praised the mayor of New York City for his efforts to institute a ban on sugary beverages, a proposed ban that has run into much opposition.^3^

So controversy continues about salt, sugar, and the annual physical. And what about exercise? Just a few days before, a front-page news story had described a study finding that some people could be “harmed” by exercise.^4^ Among 1687 patients who were enrolled in 6 separate studies of rigorous exercise, approximately 10% saw worsening of measures traditionally thought to improve with exercise; these measures included blood pressure, high-density lipoprotein cholesterol, triglycerides, and fasting insulin levels.^5^ The news story included questions about current guidelines that call for at least 150 minutes of moderate-level exercise per week. The guidelines are based on “a weak form of evidence…that compared the health of people who exercised with that of people who did not.”^4^

Within the context of these ongoing controversies that focus on our approach to fundamental lifestyle issues, Barlow and colleagues^6^ report in the current issue of the *Journal of the American Heart Association* on the association of cardiorespiratory fitness and long-term risk of death among adults classified as low risk by the Framingham Risk Score.^6^ The authors performed symptom-limited exercise tests in >11 000 healthy adults without diabetes mellitus who were followed up for 27 years, during which time >1000 died. There was a strong gradient of risk, as decreasing levels of fitness predicted increased risk of all-cause and cardiovascular death, irrespective of sex, smoking status, or baseline Framingham Risk Score. The authors argue that their findings have “important implications for clinical practice” in that low-risk adults should be considered appropriate candidates for higher doses of aerobic exercise.

Barlow and colleagues’ well-done investigation is wholly consistent with a remarkably robust body of literature on the epidemiological association between levels of cardiorespiratory fitness and risk of death. Across a wide spectrum of baseline risk, from severe heart failure to “low-risk” asymptomatic adults, lower levels of fitness predict higher rates of death. How should we respond to these findings? We can frame our responses to the literature on fitness and to Barlow and colleagues’ report within clinical, public health, and research contexts.

## Clinical implications

Barlow et al^6^ focused on patients deemed to be at low risk on the basis of the Framingham Risk Score, a global risk assessment that incorporates age, sex, smoking status, blood pressure, and cholesterol levels.^7^ Although risk scores have been promoted widely, remarkably little evidence exists to show that their routine use leads to improved clinical outcomes.^7^ Furthermore, risk scores have been criticized for yielding inconsistent values; recent work has highlighted remarkably high levels of predictive uncertainty, as levels of risk can depend not only on clinical characteristics but also on which risk instrument and risk variables are measured.^8,9^ The study by Barlow et al effectively represents a refinement of the Framingham Risk Score, a refinement based on the addition of cardiorespiratory fitness. It is difficult for us to determine how important this refinement is because the authors do not present data on improved discrimination or reclassification.^10^ And even so, the study, as well done as it is, is an observational study and therefore is subject to all the often underappreciated biases of observational epidemiology.

## Public health implications

Current US guidelines recommend that most adults seek to engage in at least moderate-level exercise for ≥150 minutes a week (eg, 30 minutes a day for 5 days a week).^4^ Barlow and colleagues argue that their data support widespread prescription of higher doses of exercise even among low-risk adults. To date, though, there are no large-scale randomized trials supporting exercise recommendations. One small trial of sedentary obese women found that as little as 72 minutes of exercise per week could lead to potentially meaningful improvements in physical fitness.^11^ A large-scale observational study of >400 000 adults suggested that even as little as 15 minutes of exercise per day predicted a 14% reduction in risk of death.^12^ Some of us worry that that people might misinterpret public health recommendations to mean that anything less than 150 minutes of exercise per week is of no value and therefore not worth pursuing at all. To add to the confusion, we now are aware of data suggesting that some adults might be harmed by exercise.^5^ It is critically important to avoid oversimplifications that overlook nuanced quantitative and qualitative issues: Just as not all fats are the same and not all carbohydrates are the same, not all exercise, whether of short or long duration, is equivalent either.

## Research implications

On the basis of what we know to date, exercise can be an extraordinarily powerful preventive intervention, an intervention that might prevent premature death, myocardial infarction, stroke, cancer, depression, dementia, and disability. Nevertheless, in contrast to the situation with blood pressure and cholesterol treatment, there is remarkably little randomized trial evidence supporting routine exercise prescription. Perhaps this is because it is much more difficult to prescribe an exercise regimen to which people will adhere than it is to prescribe pills. So far, trials have focused either on surrogate measures or on special populations, like those with heart failure^13^ or glucose intolerance.^14^ The ongoing LIFE trial (Lifestyle Interventions and Independence for Elders; ClinicalTrials.gov Identifier: NCT01072500) is assessing the value of routine exercise in sedentary healthy elderly adults, and although it is powered primarily to evaluate effects on mobility, it might yield some important insights on the effects of exercise prescription on cardiovascular health.

There also could be a role for public health research into the effects of public policies on levels of physical activity. Community planners can consider a variety of interventions, including installation of pavements and increased numbers of neighborhood recreational facilities. It is arguably even more difficult to randomize community interventions than it is to randomize clinical lifestyle interventions, though some investigators have demonstrated an ability to randomize neighborhoods^15^ or schools^16^ to various types of public health efforts. Another potentially valid research methodology involves exploitation of “natural experiments,” such as differential implementation of smoking bans across political borders.^17^

Over the past 50 years, we have seen remarkable declines in death rates due to myocardial infarction and stroke. A good part of the improvements can be traced to management of classic risk factors. The recently announced “Million Hearts Initiative” will seek to exploit 4 evidence-based interventions: aspirin in appropriate settings, blood pressure reduction, cholesterol reduction, and smoking cessation.^18^ Even if the initiative is successful in preventing millions of heart attacks and strokes, cardiovascular disease will remain a leading cause of death and disability. It is wholly appropriate that we are exploring other modes of prevention, including exercise, but at the same time we should have the humility and discipline to figure out how to develop robust levels of evidence that will enable us not only to confidently improve public health, but also to better enjoy our encounters with the Sunday newspaper.

## References

[1] Rosenthal E Let's (not) get physicals. New York Times June 3, 2012. Available at: http://www.nytimes.com/2012/06/03/sunday-review/lets-not-get-physicals.html?pagewanted=all. Accessed June 4, 2012

[2] Taubes G Salt, we misjudged you. New York Times June 3, 2012. Available at: http://www.nytimes.com/2012/06/03/opinion/sunday/we-only-think-we-know-the-truth-about-salt.html?partner=rss&emc=rss. Accessed June 4, 2012

[3] Bruni F Trimming a fat city. New York Times June 3, 2012. Available at: http://www.nytimes.com/2012/06/03/opinion/sunday/bruni-trimming-a-fat-city.html?_r=1&partner=rss&emc=rss. Accessed June 4, 2012

[4] Kolata G For some, exercise may increase heart risk. New York Times May 31, 2012. Available at: http://well.blogs.nytimes.com/2012/05/30/can-exercise-be-bad-for-you/?ref=ginakolata. Accessed June 4, 2012

[5] Bouchard C Blair SN Church TS Earnest CP Hagberg JM Häkkinen K Jenkins NT Karavirta L Kraus WE Leon AS Rao DC Sarzynski MA Skinner JS Slentz CA Rankinen T Adverse metabolic response to regular exercise: is it a rare or common occurrence?. PLoS ONE. 2012;7:e37887 22666405 10.1371/journal.pone.0037887 PMC3364277

[6] Barlow CE DeFina LF Radford NB Berry JD Cooper KH Haskell WL Jones LW Lakoski SG Cardiorespiratory fitness and long-term survival in “low-risk” adults. J Am Heart Assoc. 2012;1:e001354 doi: 10.1161/JAHA.112.001354 10.1161/JAHA.112.001354 PMC3487345 23130161

[7] Greenland P Alpert JS Beller GA Benjamin EJ Budoff MJ Fayad ZA Foster E Hlatky MA Hodgson JM Kushner FG Lauer MS Shaw LJ Smith SC Jr Taylor AJ Weintraub WS Wenger NK Jacobs AK 2010 ACCF/AHA guideline for assessment of cardiovascular risk in asymptomatic adults: a report of the American College of Cardiology Foundation/American Heart Association Task Force on Practice Guidelines. Circulation. 2010;122:e584-e636 21098428 10.1161/CIR.0b013e3182051b4c

[8] Kent DM Shah ND Risk models and patient-centered evidence: should physicians expect one right answer?. JAMA. 2012;307:1585-1586 22511683 10.1001/jama.2012.469

[9] Cook NR Paynter NP Eaton CB Manson JE Martin LW Robinson JG Rossouw JE Wassertheil-Smoller S Ridker PM Comparison of the Framingham and Reynolds Risk scores for global cardiovascular risk prediction in the multiethnic Women's Health Initiative. Circulation. 2012;125:1748-1756 22399535 10.1161/CIRCULATIONAHA.111.075929 PMC3324658

[10] Hlatky MA Greenland P Arnett DK Ballantyne CM Criqui MH Elkind MS Go AS Harrell FE Jr Hong Y Howard BV Howard VJ Hsue PY Kramer CM McConnell JP Normand SL O'Donnell CJ Smith SC Jr Wilson PW Criteria for evaluation of novel markers of cardiovascular risk: a scientific statement from the American Heart Association. Circulation. 2009;119:2408-2416 19364974 10.1161/CIRCULATIONAHA.109.192278 PMC2956982

[11] Church TS Earnest CP Skinner JS Blair SN Effects of different doses of physical activity on cardiorespiratory fitness among sedentary, overweight or obese postmenopausal women with elevated blood pressure: a randomized controlled trial. JAMA. 2007;297:2081-2091 17507344 10.1001/jama.297.19.2081

[12] Wen CP Wai JPM Tsai MK Yang YC Cheng TYD Lee M-C Chan HT Tsao CK Tsai SP Wu X Minimum amount of physical activity for reduced mortality and extended life expectancy: a prospective cohort study. Lancet. 2011;378:1244-1253 21846575 10.1016/S0140-6736(11)60749-6

[13] O'Connor CM Whellan DJ Lee KL Keteyian SJ Cooper LS Ellis SJ Leifer ES Kraus WE Kitzman DW Blumenthal JA Rendall DS Miller NH Fleg JL Schulman KA McKelvie RS Zannad F Pina IL Efficacy and safety of exercise training in patients with chronic heart failure: HF-ACTION randomized controlled trial. JAMA. 2009;301:1439-1450 19351941 10.1001/jama.2009.454 PMC2916661

[14] Knowler WC Barrett-Connor E Fowler SE Hamman RF Lachin JM Walker EA Nathan DM Reduction in the incidence of type 2 diabetes with lifestyle intervention or metformin. N Engl J Med. 2002;346:393-403 11832527 10.1056/NEJMoa012512 PMC1370926

[15] Jafar TH Hatcher J Poulter N Islam M Hashmi S Qadri Z Bux R Khan A Jafary FH Hameed A Khan A Badruddin SH Chaturvedi N Community-based interventions to promote blood pressure control in a developing country. Ann Internal Med. 2009;151:593-601 19884620 10.7326/0003-4819-151-9-200911030-00004

[16] Klesges RC Obarzanek E Kumanyika S Murray DM Klesges LM Relyea GE Stockton MB Lanctot JQ Beech BM McClanahan BS Sherrill-Mittleman D Slawson DL The Memphis Girls’ health Enrichment Multi-site Studies (GEMS): an evaluation of the efficacy of a 2-year obesity prevention program in African American girls. Arch Pediatr Adolesc Med. 2010;164:1007-1014 21041593 10.1001/archpediatrics.2010.196 PMC3052791

[17] Pell JP Haw S Cobbe S Newby DE Pell ACH Fischbacher C McConnachie A Pringle S Murdoch D Dunn F Oldroyd K MacIntyre P O'Rourke B Borland W Smoke-free legislation and hospitalizations for acute coronary syndrome. N Engl J Med. 2008;359:482-491 18669427 10.1056/NEJMsa0706740

[18] Frieden TR Berwick DM The “Million Hearts” initiative–preventing heart attacks and strokes. N Engl J Med. 2011;365:e27 21913835 10.1056/NEJMp1110421

